# Seven routine blood markers predict five-year mortality in older Indians: a risk score from the LASI-DAD cohort

**DOI:** 10.21203/rs.3.rs-9979288/v1

**Published:** 2026-07-09

**Authors:** Bhrigu Jain, Sharmistha Dey, Aparajit Dey

**Affiliations:** All India Institute of Medical Sciences; All India Institute of Medical Sciences; Artemis Hospitals

**Keywords:** Mortality, Risk prediction, Biomarkers, Older adults, India, LASI-DAD, Frailty, Reverse epidemiology

## Abstract

**Background.:**

Validated, blood-based tools to estimate short-to-medium-term mortality in older adults are derived almost entirely from European-ancestry or East Asian populations and transport poorly to South Asians, who present a distinct cardiometabolic and body-composition phenotype. No mortality risk score using routine blood markers exists for community-dwelling older Indians.

**Methods.:**

We used Wave-1 data from the Longitudinal Aging Study in India – Diagnostic Assessment of Dementia (LASI-DAD), a nationally representative cohort of adults aged ≥ 60 years with venous biomarker phenotyping. Vital status was ascertained through an End-of-Life informant module to Wave 2. From the available venous panel, we pre-selected seven markers on biological grounds — N-terminal pro-B-type natriuretic peptide (NT-proBNP), serum albumin, cystatin C, high-sensitivity C-reactive protein (hs-CRP), the neutrophil-to-lymphocyte ratio (NLR), glycated haemoglobin (HbA1c) and alanine aminotransferase (ALT) — and fitted a Cox proportional-hazards model with age and sex. Discrimination (Harrell’s C), bootstrap optimism correction, calibration at three years, and risk-tertile stratification were assessed; decision-curve analysis quantified clinical utility.

**Results.:**

Among 2144 participants (540 deaths over a median 4.4 years of survivor follow-up; 25% mortality), the seven-marker score plus age and sex achieved an apparent C of 0.74 (optimism-corrected 0.74), versus 0.68 for age and sex alone (Δ ≈ 0.06); the blood panel alone reached C 0.72. NT-proBNP (hazard ratio [HR] per 1 SD 1.31), HbA1c (1.24) and albumin (0.81) carried most of the signal, with smaller contributions from cystatin C, ALT, NLR and hs-CRP. Calibration was excellent at three years. Participants in the highest score tertile had 8.7-fold the mortality hazard of the lowest (95% CI 6.5–11.6; log-rank p < 0.001), with three-year mortality of 34% versus 6%. The score provided positive net benefit across clinically relevant decision thresholds. In sensitivity analyses, all associations persisted after adjustment for comorbidity, cognition and function, and the panel out-performed a clinical-plus-cognitive model.

**Conclusions.:**

A seven-marker panel obtainable from a single venous sample, combined with age and sex, stratifies five-year mortality in older Indians with good discrimination and calibration. Pending external validation, it offers a clinically translatable, population-specific tool for prognostic risk stratification in a large and under-studied population.

**Trial registration:**

Not applicable (observational cohort analysis).

## Background

India is home to the world’s second-largest population of adults aged 60 years and older, a group projected to exceed 230 million by 2036. Identifying who among them is at greatest risk of dying in the next few years is fundamental to prioritising geriatric assessment, targeting interventions, and enriching clinical trials, yet the tools available to do so were not built for this population. Widely used prognostic instruments such as the Pooled Cohort Equations, Framingham, QRISK and the biomarker-based PhenoAge and Intermountain Risk Score were derived in European-ancestry or, more recently, East Asian cohorts [1–3], and they systematically miscalibrate when transported to South Asians. South Asians develop diabetes earlier and at lower body-mass index, carry more visceral and less skeletal muscle mass at any given weight (the “thin-fat” phenotype), have a higher background burden of chronic inflammation and anaemia, and experience excess cardiovascular risk that conventional calculators underestimate [4–8]. A mortality risk score derived in a representative Indian population, using markers obtainable from any district-hospital laboratory, would address a clear translational gap.

LASI-DAD is a nationally representative cohort of community-dwelling Indians aged ≥ 60 with deep biomarker, cognitive and genomic phenotyping at Wave 1 and active follow-up through Wave 2, including an End-of-Life informant module that ascertains deaths occurring between waves [9–10]. Approximately one in four Wave-1 participants died over the roughly five-year interwave interval [10], yielding ample events for prognostic modelling. Prior biomarker work in this cohort has been informative but has stopped short of a prognostic score. Hu and colleagues established cross-sectional associations between cardiometabolic-inflammatory markers and cognition [11]; Kim and colleagues characterised plasma neurodegenerative markers and cognition [12]; and Banerjee and colleagues recently reported individual-marker associations with between-wave death using logistic regression, notably NT-proBNP and serum albumin, but did not combine markers into a unified score, did not use time-to-event modelling, and reported no discrimination or calibration metrics [13]. Rao and colleagues demonstrated that the WHO intrinsic-capacity construct predicts survival in LASI-DAD, the first Cox mortality model in this cohort, but used clinical and functional domains rather than blood biomarkers [14].

Internationally, multi-biomarker mortality models have a strong track record. The Intermountain Risk Score reaches a C-statistic of 0.85–0.90 from complete-blood-count and metabolic-panel inputs in clinical-laboratory populations [1]; PhenoAge, built from nine routine biomarkers, predicts mortality across diverse subgroups [2–3]; the ULSAM four-marker score (NT-proBNP, cystatin C, hs-CRP, troponin I) reaches C ≈ 0.71 in older men [15]; and a seven-routine-laboratory score in the Leiden 85-Plus Study reached C = 0.66 in the oldest old [16]. Omics-based scores in UK Biobank and Chinese cohorts exceed C = 0.80 but rely on platforms unavailable in routine Indian care [17–19]. None has been developed in South Asians.

A further consideration shaped our approach. In older adults the relationship between routine laboratory markers and death is frequently not the one clinicians carry over from midlife. Several established “risk factors” reverse direction with age: low rather than high total cholesterol, low rather than high blood pressure, and low rather than high body weight predict mortality, because in late life these values tend to mark frailty, undernutrition and occult illness rather than cardiovascular protection, a pattern widely termed reverse epidemiology [20–21]. Markers of physiological reserve behave the same way, with low albumin, haemoglobin, muscle-derived aminotransferases and thyroid hormone flagging depleted reserve rather than health. These paradoxes are likely to be especially pronounced in older Indians, in whom undernutrition, anaemia and sarcopenia are highly prevalent. We therefore screened the entire venous panel against mortality rather than restricting attention to canonical risk factors, letting the data show which markers, and in which direction, carry prognostic weight in this population.

We therefore set out to develop and internally validate a parsimonious, blood-based, multi-marker mortality risk score in LASI-DAD. We pre-specified seven markers spanning cardiac stress (NT-proBNP), renal function (cystatin C), nutritional and hepatic reserve (albumin, ALT), systemic inflammation (hs-CRP, NLR) and glycaemic burden (HbA1c). We hypothesised that a score built from these markers would discriminate mortality substantially beyond age and sex, would be well calibrated, and would expose biology specific to this population.

## Methods

### Study population and design

LASI-DAD is a nationally representative sub-study of the Longitudinal Aging Study in India, enrolling adults aged ≥ 60 years for in-depth cognitive and biomarker assessment [9]. Wave-1 assessments (2017–2020) included a venous blood draw analysed for a panel of cardiac, renal, hepatic, inflammatory, haematological, endocrine and nutritional markers. This analysis follows the TRIPOD reporting framework for prediction-model development and validation [22].

### Outcome and follow-up

The outcome was all-cause mortality. Deaths occurring after the Wave-1 assessment were ascertained through the LASI-DAD End-of-Life informant module administered at Wave 2, which records the timing of death; surviving participants reassessed at Wave 2 contributed observed follow-up time [10]. Time at risk was measured from the Wave-1 assessment. For decedents, survival time was the recorded interval from Wave-1 assessment to death; for survivors, it was the interval from the Wave-1 to the Wave-2 assessment. Participants alive but not reassessed at Wave 2 were treated as lost to follow-up and excluded from the time-to-event analysis. Because all ascertained deaths occurred before the Wave-2 assessment, the score was anchored to Wave-1 biomarkers; analytes measured only at Wave 2 were ineligible as baseline predictors, and within-person biomarker change could not be evaluated. The prospective interval from the Wave-1 assessment to death constitutes the longitudinal dimension of the analysis.

### Candidate markers and the final panel

**Figure 1:**
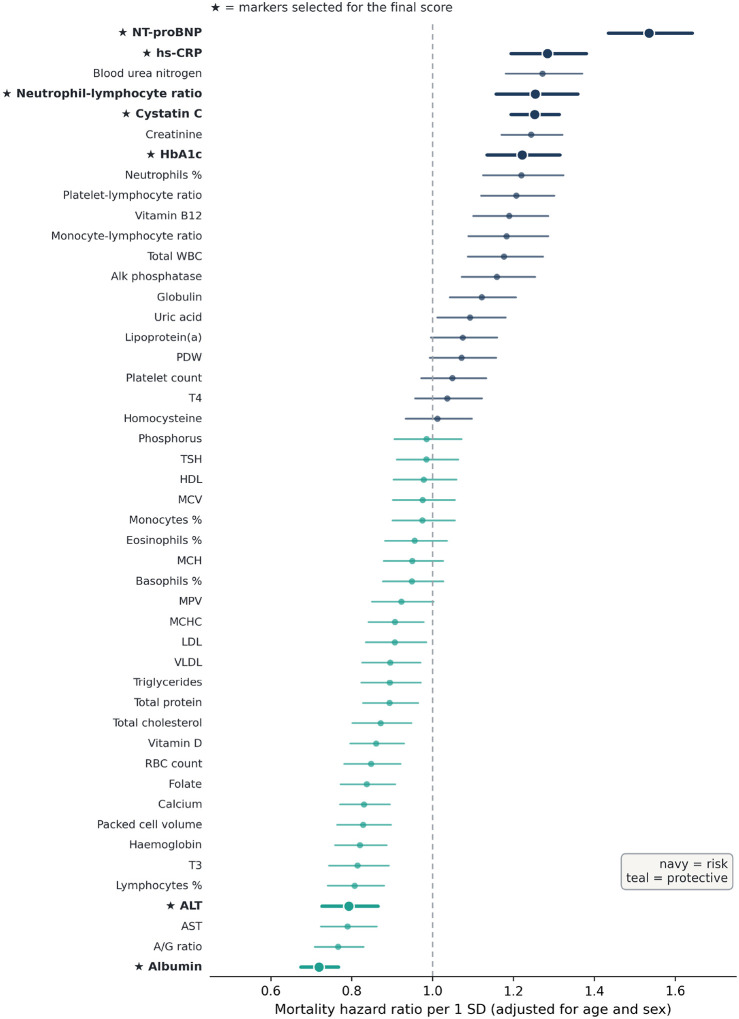
Univariate mortality screen of the full blood marker panel. Age- and sex-adjusted hazard ratios per 1 SD for every measured marker, ranked by effect size; starred markers were selected for the final score. Navy denotes risk-increasing, teal risk-decreasing associations.

From the full Wave-1 venous panel we first screened every measured marker against mortality in age-and sex-adjusted Cox models (Supplementary Table S1; Fig. 1). Guided by this screen and by prior biology, and to avoid redundancy within correlated biological axes (e.g., cystatin C, creatinine and blood urea nitrogen all index renal filtration; haemoglobin, packed-cell volume and red-cell count index anaemia), we pre-specified a parsimonious seven-marker panel with one representative per axis: NT-proBNP (cardiac stress), cystatin C (renal function), serum albumin and ALT (nutritional and hepatic/muscle-mass reserve), hs-CRP and the neutrophil-to-lymphocyte ratio (systemic inflammation), and HbA1c (glycaemic burden). Right-skewed markers (NT-proBNP, cystatin C, hs-CRP, NLR, HbA1c, ALT) were natural-log-transformed; all markers were standardised to 1-SD units so that hazard ratios are comparable across markers. The NLR was computed from the differential leukocyte count.

### Statistical analysis

Baseline characteristics are summarised as mean (SD) or median [IQR] for continuous variables and n (%) for categorical variables, and compared between survivors and decedents using t or Wilcoxon rank-sum tests and the chi-squared test (Table 1). We fitted a multivariable Cox proportional-hazards model with the seven standardised markers plus age (per 1 SD) and sex, reporting hazard ratios per 1-SD increment with 95% confidence intervals. Discrimination was quantified by Harrell's C, with optimism estimated from 200 bootstrap resamples and the optimism-corrected C reported; the full model was compared with age-and-sex-only and biomarkers-only models. Calibration was assessed by comparing predicted (Breslow baseline hazard) with observed (Kaplan–Meier) three-year mortality across deciles and tertiles of risk. Participants were grouped into tertiles of the linear predictor (log-rank test; high-versus-low tertile hazard ratio), and clinical utility was assessed by decision-curve analysis at three years against age-and-sex and the treat-all/treat-none strategies; the full model specification, enabling individual risk estimation, is provided as a coefficient equation (Supplementary Table S3) and an interactive calculator (Additional file 4). In a sensitivity analysis, the model was re-estimated with additional adjustment for comorbidity burden (Hypertension, Diabetes, Heart disease, Stroke, Chronic lung disease and Cancer), baseline cognition (HMSE) and function (any ADL/IADL difficulty) on a common complete-case sample.

No formal power calculation was performed, as is standard for prediction-model development; with 540 events and nine parameters the events-per-variable ratio (~ 60) was well above thresholds for stable estimation. Analyses used Python (version 3.12), with Cox estimation, concordance, Kaplan–Meier and log-rank procedures implemented directly; the proportional-hazards assumption was assessed using scaled Schoenfeld residuals (Grambsch–Therneau global and per-covariate tests). Generative AI assistance was used for code drafting and language editing under author supervision, with all analyses and interpretations verified by the authors; large language models do not meet authorship criteria and are not listed as authors.

## Results

### Cohort characteristics

The analytic sample comprised 2144 participants with the complete seven-marker panel and valid follow-up. Over a median survivor follow-up of 4.4 years, 540 participants (25.2%) died; median time from assessment to death among decedents was 2.2 years. Compared with survivors, decedents were older (mean 73.9 vs 68.3 years), more often male, and leaner (BMI 21.3 vs 22.7 kg/m^2^), and carried a heavier comorbidity burden, more diabetes (22.2% vs 13.7%), self-reported hypertension (43.4% vs 37.8%), prior stroke (6.2% vs 2.2%) and heart disease (Table 1). They differed across every component of the biomarker panel, higher NT-proBNP, cystatin C, hs-CRP, NLR, blood urea nitrogen, uric acid and homocysteine, and lower albumin, haemoglobin, total cholesterol, triiodothyronine, ALT and cystatin C-based eGFR. Decedents also had markedly worse baseline cognition and function: lower HMSE and general cognition scores, a higher prevalence of dementia (15.9% vs 5.4%) and MCI (22.8% vs 16.2%), and more frequent ADL (61.6% vs 49.7%) and IADL (75.5% vs 66.6%) difficulty (all p < 0.001). Educational attainment did not differ significantly by vital status.

### Univariate associations across the full panel

Before model building, we screened every measured venous marker against mortality in age- and sex-adjusted models (Fig. 1; Supplementary Table S1). Thirty-three of 47 markers were significantly associated with death, and the associations fell into three coherent biological groups. In the first, higher values signalled higher risk: the cardiac and renal markers (NT-proBNP, cystatin C, creatinine, blood urea nitrogen, uric acid) and the inflammatory markers (hs-CRP, the neutrophil-, monocyte- and platelet-to-lymphocyte ratios, total white-cell count). A second and larger group ran in the opposite direction, lower values signalled higher risk, and consisted almost entirely of markers of nutritional, hepatic, haematological and endocrine reserve: albumin and the albumin-to-globulin ratio, ALT and AST, triiodothyronine, haemoglobin and the other red-cell indices, total cholesterol and its fractions, vitamin D, folate and calcium. A third set carried no mortality signal, including homocysteine (HR 1.01 per SD, p = 0.77), thyroid-stimulating hormone, HDL cholesterol and most red-cell morphology indices. One marker was frankly paradoxical: vitamin B12, where higher concentrations predicted higher mortality (HR 1.19, p < 0.001). This screen motivated a score that draws one representative from each informative biological axis rather than stacking correlated markers, and that deliberately omits markers (such as B12) whose direction would mislead at the bedside.

### Multivariable model

**Figure 2:**
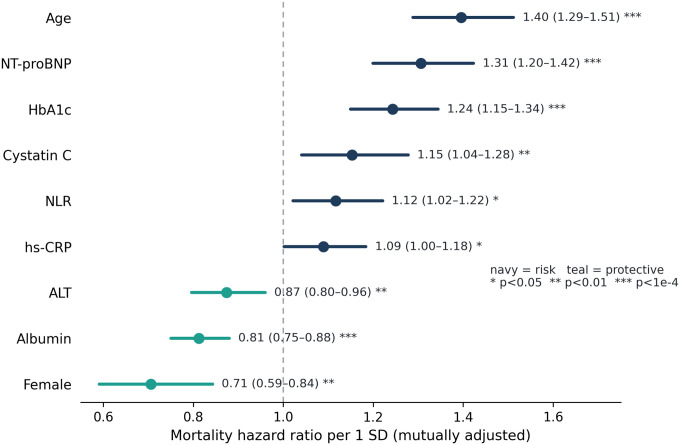
Multivariable mortality model. Forest plot of adjusted hazard ratios per 1 SD for the seven markers plus age and sex; navy denotes risk-increasing and teal risk-decreasing associations.

In the mutually adjusted model (Table 2, Fig. 2), all seven markers were independently associated with mortality. NT-proBNP showed the strongest biomarker association (HR per 1 SD 1.31, 95% CI 1.20–1.42), followed by HbA1c (1.24, 1.15–1.34) and lower albumin (0.81, 0.75–0.88). Cystatin C (1.15, 1.04–1.28), ALT (lower; 0.87, 0.80–0.96), NLR (1.12, 1.02–1.22) and hs-CRP (1.09, 1.00–1.18) each contributed smaller but significant independent signal. Age remained the single strongest predictor (1.40 per 1 SD, 1.29–1.51) and female sex was protective (0.71, 0.59–0.84). The relative magnitudes of the hazard ratios indicate that NT-proBNP, HbA1c and albumin together account for the majority of the biomarker signal.

### Discrimination and calibration

**Figure 3:**
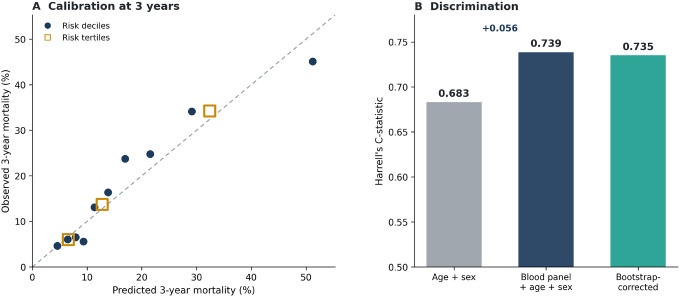
Calibration and discrimination. (A) Predicted versus observed three-year mortality across deciles (circles) and tertiles (squares) of predicted risk, with the identity line. (B) Harrell’s C for age and sex alone, the full model, and the bootstrap optimism-corrected estimate.

The full model achieved an apparent Harrell’s C of 0.739; bootstrap optimism was negligible (0.004), giving an optimism-corrected C of 0.735. This compared with 0.683 for age and sex alone, an increment of 0.056 attributable to the blood panel, and 0.72 for the biomarkers without age and sex, indicating that the markers carry most of the prognostic information. Calibration at three years was excellent, with predicted and observed mortality closely aligned across deciles and tertiles of predicted risk (Fig. 3). Proportional hazards assumption was satisfied

### Risk stratification and clinical utility

**Figure 4:**
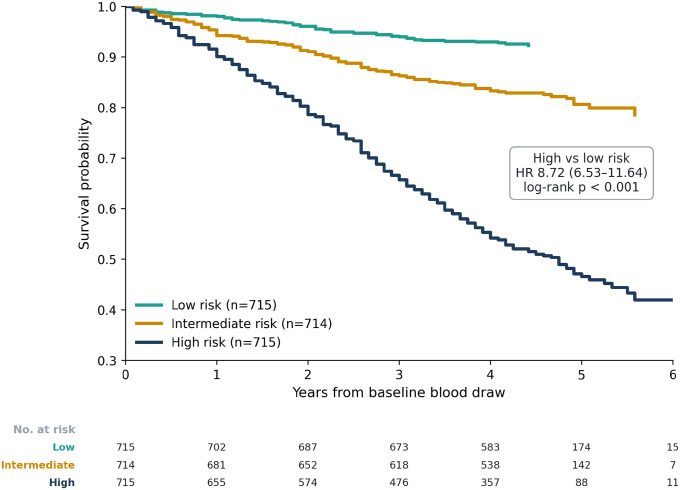
Survival by blood marker-score risk tertile. Kaplan–Meier survival curves for the low, intermediate and high tertiles of the seven-marker score, with numbers at risk. The highest tertile had 8.7-fold the mortality hazard of the lowest (95% CI 6.5–11.6; log-rank p<0.001).

**Figure 5:**
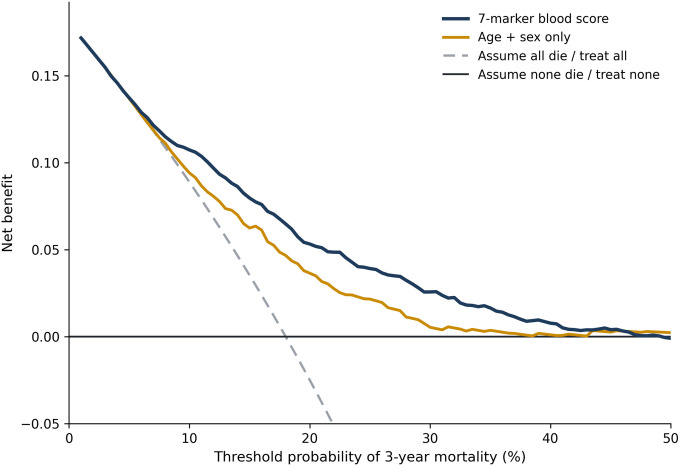
Decision-curve analysis at three years. Net benefit of the seven-marker score, age and sex alone, and the default treat-all and treat-none strategies across threshold probabilities of three-year mortality.

Tertiles of the score separated survival sharply (Fig. 4; log-rank p < 0.001). Participants in the highest-risk tertile had 8.7-fold the mortality hazard of those in the lowest (95% CI 6.5–11.6), corresponding to observed three-year mortality of approximately 34% in the highest tertile, 14% in the middle, and 6% in the lowest. In decision-curve analysis at three years, the score provided greater net benefit than both default strategies and than age and sex alone across the full range of clinically relevant threshold probabilities (Fig. 5), supporting potential clinical utility for triage and risk-based prioritisation.

### Sensitivity analysis

The seven biomarker associations were robust to further adjustment. In a model additionally including comorbidity burden, baseline cognition (HMSE) and functional status (any activities-of-daily-living and instrumental-activities-of-daily-living difficulty), all seven markers remained individually significant with essentially unchanged estimates (NT-proBNP HR 1.24, albumin 0.84, HbA1c 1.22, cystatin C 1.20, ALT 0.88, NLR 1.11, hs-CRP 1.10; all p < 0.05; Supplementary Table S2). On a common sample, the biomarker panel improved discrimination over a model containing age, sex, comorbidity, cognition and function (Harrell’s C 0.748 vs 0.699), and the blood-only score alone out-discriminated that clinical-plus-cognitive model (C 0.739 vs 0.699), indicating that the markers carry prognostic information not captured by clinical history or by cognitive and functional assessment. Results were materially unchanged when comorbidity was modelled as six individual conditions rather than as a count.

## Discussion

A seven-marker blood panel, combined with age and sex, stratified five-year all-cause mortality in a nationally representative sample of community-dwelling older Indians with an optimism-corrected C of 0.74 and an 8.7-fold hazard contrast between the highest and lowest risk tertiles. To our knowledge, this is the first internally validated, blood-based, multi-marker mortality risk score developed in a South Asian aging cohort, and one of only two formal time-to-event mortality models published in LASI-DAD, the other being the intrinsic-capacity analysis of Rao and colleagues, which used functional rather than biochemical predictors [14]. Our work adds biological interpretability and laboratory translatability to mortality risk stratification in this population.

### Comparison with existing scores.

Our discrimination sits within the expected range for routine-laboratory mortality models in community-dwelling older adults. It exceeds the most direct design analogue, the Leiden 85-Plus seven-routine-laboratory score (C = 0.66 at age 85) [16], and is comparable to the ULSAM four-marker biomarker score (C ≈ 0.71), with which we share three markers (NT-proBNP, cystatin C, hs-CRP) [15]. It is lower than the Intermountain Risk Score (C = 0.85–0.90), which was derived in clinical-laboratory populations enriched for acute illness and uses a larger panel [1], and lower than omics-based scores in UK Biobank and CHARLS (C ≈ 0.80) that rely on metabolomic platforms not yet available in routine Indian care [17–19]. The incremental gain of the blood panel over age and sex (≈ 0.06 in C) is modest in absolute terms, as it is in most biomarker-addition studies in older populations [23], because chronological age dominates mortality prediction at these ages; the markers nonetheless add information that is both statistically robust and biologically actionable.

### Biological interpretation.

Each marker recapitulates established biology. NT-proBNP, the dominant biomarker, reflects subclinical cardiac and renal stress and is a well-established mortality predictor in older adults [24]; its magnitude here is concordant with the within-cohort findings of Banerjee and colleagues [13]. Cystatin C tracks subclinical kidney dysfunction [25]; hs-CRP and NLR index systemic inflammation [26–27]; and lower albumin reflects undernutrition, inflammation and depleted synthetic reserve [28]. The HbA1c association (HR 1.24 per SD) is somewhat larger than reported in European cohorts [29], plausibly reflecting the South Asian diabetes phenotype of earlier onset at lower BMI and higher glycaemia for a given adiposity [8, 30].

The most distinctive finding is the inverse ALT association: lower ALT predicted higher mortality (HR 0.87 per SD), opposite to the metabolic narrative in which elevated aminotransferases mark hepatic injury. This replicates a consistent body of work in older European cohorts re-framing low ALT as a marker of low muscle mass, frailty and sarcopenia rather than of hepatic health [31–33]. In a population where sarcopenia affects more than 40% of older adults [34] and the thin-fat, low-lean-mass phenotype is established from early life [5], this signal is biologically expected and clinically meaningful. To our knowledge this is its first demonstration in a South Asian cohort.

### Significant, null and paradoxical markers — reverse epidemiology in late life.

The full-panel screen is itself instructive, and several of its patterns run counter to midlife clinical intuition in ways worth spelling out. The most conspicuous is the large cluster of markers in which low, not high, values predicted death — albumin, ALT and AST, triiodothyronine, haemoglobin and total cholesterol. This is the now wellrecognised phenomenon of reverse epidemiology, in which exposures that are hazardous in midlife invert in old age because low values come to mark frailty, undernutrition, inflammation and occult disease rather than good health [20–21]. Low total cholesterol is the clearest example: it predicted higher mortality (HR 0.87 per SD) not because cholesterol protects, but because hypocholesterolaemia in older people signals poor nutritional status, chronic illness and the catabolic state that precedes death [20]. We make this interpretation explicit to forestall the misreading that high cholesterol is beneficial here. The low-aminotransferase and low-triiodothyronine signals belong to the same family, low ALT and AST index reduced muscle mass and sarcopenia [31–33], and low T3 reflects the non-thyroidal-illness (“sick-euthyroid”) response to systemic disease.

Anaemia deserves specific comment. Low haemoglobin was strongly associated with mortality on univariate screening (HR 0.82 per SD), consistent with the heavy anaemia burden in older Indians [35] and with anaemia’s established prognostic weight in ageing populations. Yet haemoglobin, like red-cell count, triiodothyronine and several other reserve markers, lost independent significance once albumin, cystatin C and the inflammatory markers were in the model, indicating that its prognostic information is largely shared with that nutritional–inflammatory–renal axis rather than standing apart from it. This is precisely why a parsimonious score already containing albumin and the inflammatory markers gains little by adding haemoglobin, even though anaemia remains unquestionably important clinically; statistical redundancy is not clinical irrelevance, and we are careful to frame it as such.

Two further findings are illuminating. Homocysteine, a marker with a large literature in vascular disease and cognition, was flatly null for mortality in this cohort (HR 1.01, p = 0.77); in LASI-DAD its relevance appears to lie with cognitive rather than survival endpoints. And vitamin B12 was genuinely paradoxical, higher concentrations predicting higher mortality (HR 1.19). Rather than implying harm from the vitamin, this most plausibly reflects reverse causation and confounding, supplementation in frail or symptomatic elders, and elevated B12 as a recognised marker of occult hepatic and malignant disease, a pattern now reported in several general-population cohorts [36]. We therefore excluded B12 from the score despite its statistical significance, because its direction would mislead clinicians who reasonably regard higher B12 as benign. Taken together, these patterns underline why we screened and interpreted the whole panel rather than a shortlist of canonical risk factors: in older adults, both the presence and the *direction* of an association carry information, and several of the most statistically striking signals are prognostic markers of depleted reserve rather than modifiable causes of death.

### Robustness.

The score’s prognostic value did not depend on the exclusion of clinical and cognitive information. Adjusting for comorbidity burden, baseline cognition and functional status left every biomarker association essentially unchanged, and the panel continued to improve discrimination beyond a model built from those clinical and cognitive variables (Additional file: Table S2). That a score derived from a single venous sample out-performed a clinical-plus-cognitive model reinforces its potential value in settings where structured geriatric assessment is not feasible.

### Population-specific relevance.

Because the score’s weights and thresholds are estimated from a representative Indian cohort, it side-steps the calibration drift that affects Western tools transported to South Asians [4, 7]. The amplified glycaemic signal and the low-ALT-as-sarcopenia inversion are consistent with the population-specific biology summarised in international consensus statements [4] and reinforce the case for population-tailored prognostication.

### Strengths and limitations.

Strengths include a nationally representative sampling frame, a standardised central biomarker panel, an interpretable parsimonious model spanning distinct biological axes, and adherence to TRIPOD reporting. Several limitations temper interpretation. First, and most importantly, validation is by internal bootstrap only; no independent South Asian cohort with comparable biomarker phenotyping is currently available, and external validation in LASI Wave-2 venous-blood subsamples, CARRS, or SAGE-India is the essential next step before clinical deployment. Second, the venous-blood subsample is a subset of the full LASI-DAD cohort, and although baseline characteristics were comparable, selection effects cannot be entirely excluded. Third, three markers initially associated with mortality (haemoglobin, triiodothyronine) lost significance under mutual adjustment and were excluded; in a population with very high anaemia prevalence this redundancy warrants confirmation. Fourth, mortality was ascertained by informant report, which is robust for all-cause death but precludes reliable cause-specific analysis. Fifth, samples were non-fasting, so fasting-dependent analytes (glucose, lipids) were not used; HbA1c and NT-proBNP are not meaningfully affected. Finally, iron-status markers (ferritin, transferrin saturation, total iron-binding capacity) were assayed only at Wave 2; because every ascertained death preceded Wave 2, these could not be evaluated as baseline predictors, and the same structure precluded modelling of within-person biomarker trajectories, so the score rests on a single Wave-1 measurement.

### Implications.

A seven-marker panel deployable in any Indian district-hospital laboratory stratified older adults into risk tertiles differing 8.7-fold in five-year hazard, with positive net benefit across clinically relevant decision thresholds. This provides a basis for risk-based prioritisation of comprehensive geriatric assessment, targeted intervention (sarcopenia screening, evaluation of NT-proBNP–implicated subclinical cardiac disease, glycaemic optimisation), and clinical-trial enrichment in resource-constrained settings. External validation, and head-to-head and combined evaluation against the intrinsic-capacity approach, are the priorities for future work and may yield a fused functional-plus-biomarker tool with superior performance.

## Conclusions

In a nationally representative cohort of older Indians, a parsimonious blood-based score combining seven routine markers with age and sex discriminated and calibrated five-year mortality well and separated risk tertiles by nearly nine-fold. Pending external validation, it offers a clinically translatable, population-specific instrument for mortality risk stratification in a large and under-studied population, and surfaces a biologically distinctive low-ALT (low-muscle-mass) signal of particular relevance to South Asian aging.

## Supplementary Files

This is a list of supplementary files associated with this preprint. Click to download.

- Additionalfile1TableS1.docx
- Additionalfile2TableS2.docx
- Additionalfile3TableS3.docx
- Additionalfile4Calculator.html
- TRIPODchecklist.docx

## Figures and Tables

**Table 1:** Baseline characteristics of the analytic cohort, overall and by vital status at follow-up.

Characteristic	Overall (n = 2144)	Survivors (n = 1604)	Decedents (n = 540)	P value
Age, mean (SD), years	69.7 (7.5)	68.3 (6.5)	73.9 (8.8)	< 0.001
Female, n (%)	1125 (52.5)	895 (55.8)	230 (42.6)	< 0.001
Education, n (%)				0.156
Less than upper secondary	1654 (77.1)	1226 (76.4)	428 (79.3)	
Upper secondary / vocational	412 (19.2)	313 (19.5)	99 (18.3)	
Tertiary	78 (3.6)	65 (4.1)	13 (2.4)	
Body-mass index, mean (SD), kg/m^2^	22.3 (4.9)	22.7 (4.9)	21.3 (4.8)	< 0.001
Systolic BP, mean (SD), mmHg	136.9 (22.7)	136.2 (21.8)	139.0 (25.1)	0.020
Diastolic BP, mean (SD), mmHg	82.3 (12.2)	82.5 (11.9)	81.6 (13.3)	0.171
Measured BP ≥ 140/90, n (%)	956 (45.2)	699 (44.0)	257 (48.7)	0.072
Hypertension (ever, self-report), n (%)	838 (39.2)	606 (37.8)	232 (43.4)	0.026
Diabetes (self-report), n (%)	338 (15.8)	219 (13.7)	119 (22.2)	< 0.001
Any heart disease, n (%)	136 (6.4)	92 (5.7)	44 (8.2)	0.053
Prior stroke, n (%)	69 (3.2)	36 (2.2)	33 (6.2)	< 0.001
Current smoker, n (%)	372 (17.4)	264 (16.5)	108 (20.2)	0.061
Current drinker, n (%)	155 (7.3)	121 (7.6)	34 (6.4)	0.403
**Biomarkers**					
NT-proBNP, median [IQR], pg/mL	144 [77–309]	124 [68–233]	284 [126–597]	< 0.001
Cystatin C, mean (SD), mg/L	1.2 (0.5)	1.2 (0.4)	1.5 (0.6)	< 0.001
eGFR-cystatin, mean (SD), mL/min/1.73m^2^	63.4 (49.0)	66.6 (47.9)	53.7 (51.0)	< 0.001
Haemoglobin, mean (SD), g/dL	12.6 (1.9)	12.8 (1.8)	12.3 (2.2)	< 0.001
Albumin, mean (SD), g/dL	4.1 (0.3)	4.2 (0.3)	4.0 (0.4)	< 0.001
Total cholesterol, mean (SD), mg/dL	183.3 (42.3)	186.4 (41.5)	174.2 (43.1)	< 0.001
Triiodothyronine (T3), mean (SD), ng/dL	91.6 (21.5)	93.5 (19.0)	85.9 (26.8)	< 0.001
Homocysteine, median [IQR], μmol/L	18.9 [14.4–26.7]	18.5 [14.2–26.1]	19.7 [15.0–28.6]	0.003
hs-CRP, median [IQR], mg/L	1.7 [0.7–4.2]	1.6 [0.7–3.7]	2.2 [0.9–6.5]	< 0.001
HbA1c, mean (SD), %	6.2 (1.5)	6.2 (1.4)	6.4 (1.8)	0.002
ALT, median [IQR], U/L	16 [12–21]	17 [13–22]	14 [10–19]	< 0.001
Neutrophil-lymphocyte ratio, median [IQR]	2.1 [1.5–2.9]	2.0 [1.5–2.8]	2.3 [1.6–3.5]	< 0.001
Uric acid, mean (SD), mg/dL	4.9 (1.4)	4.8 (1.3)	5.2 (1.4)	< 0.001
Blood urea nitrogen, mean (SD), mg/dL	12.8 (5.4)	12.1 (3.8)	15.0 (8.2)	< 0.001
Creatinine, median [IQR], mg/dL	0.8 [0.7–0.9]	0.8 [0.7–0.9]	0.9 [0.7–1.1]	< 0.001
**Cognition and function**					
HMSE total, mean (SD) (0–30)	22.5 (5.4)	23.1 (4.8)	20.8 (6.4)	< 0.001
General cognition factor (z), mean (SD)	−0.1 (0.9)	0.0 (0.9)	−0.4 (1.0)	< 0.001
DSM-5 status, n (%)				< 0.001
Normal cognition	1589 (74.1)	1258 (78.4)	331 (61.3)	
MCI	383 (17.9)	260 (16.2)	123 (22.8)	
Dementia	172 (8.0)	86 (5.4)	86 (15.9)	
Any ADL difficulty, n (%)	1127 (52.7)	797 (49.7)	330 (61.6)	< 0.001
Any IADL difficulty, n (%)	1472 (68.8)	1068 (66.6)	404 (75.5)	< 0.001

Values are mean (SD) for normally distributed continuous variables, median [IQR] for skewed continuous variables, and n (%) for categorical variables. Survivors and decedents were compared using Welch's t-test (normally distributed), the Wilcoxon rank-sum test (skewed) or Pearson's chi-squared test (categorical); all p-values are two-sided, with significance at p < 0.05. eGFR was estimated using the 2012 CKD-EPI cystatin C equation. Abbreviations: ADL activities of daily living; ALT alanine aminotransferase; BP blood pressure; CKD-EPI Chronic Kidney Disease Epidemiology Collaboration; DSM-5 Diagnostic and Statistical Manual of Mental Disorders, 5th edition; eGFR estimated glomerular filtration rate; HbA1c glycated haemoglobin; HMSE Hindi Mental State Examination; hs-CRP high-sensitivity C-reactive protein; IADL instrumental activities of daily living; IQR interquartile range; MCI mild cognitive impairment; NLR neutrophil-to-lymphocyte ratio; NT-proBNP N-terminal pro-B-type natriuretic peptide; SD standard deviation.

**Table 2:** Multivariable Cox model for all-cause mortality.

Predictor	HR (95% CI) per 1 SD	P value
Age	1.40 (1.29–1.51)	< 0.001
Female sex	0.71 (0.59–0.84)	< 0.001
NT-proBNP	1.31 (1.20–1.42)	< 0.001
HbA1c	1.24 (1.15–1.34)	< 0.001
Albumin	0.81 (0.75–0.88)	< 0.001
Cystatin C	1.15 (1.04–1.28)	0.004
ALT	0.87 (0.80–0.96)	0.004
NLR	1.12 (1.02–1.22)	0.015
hs-CRP	1.09 (1.00–1.18)	0.045

Hazard ratios (HR) with 95% confidence intervals (CI) are expressed per 1-SD increment for continuous predictors (NT-proBNP, cystatin C, hs-CRP, NLR, HbA1c and ALT natural-log-transformed before standardisation) and as female versus male for sex. All predictors were entered simultaneously, so each HR is mutually adjusted for every other variable in the table. p-values are two-sided (Wald test); significance at p < 0.05. Model discrimination: apparent Harrell's C = 0.739, bootstrap optimism-corrected C = 0.735 (200 resamples). Based on 2144 participants and 540 deaths. *Abbreviations*: ALT alanine aminotransferase; C concordance statistic; CI confidence interval; HbA1c glycated haemoglobin; HR hazard ratio; hs-CRP high-sensitivity C-reactive protein; NLR neutrophil-to-lymphocyte ratio; NT-proBNP N-terminal pro-B-type natriuretic peptide; SD standard deviation.

## Data Availability

The harmonised LASI-DAD datasets analysed in the current study are available to approved researchers through the Gateway to Global Aging Data (https://g2aging.org) and the LASI-DAD study website (https://lasi-dad.org), subject to registration and a data-use agreement. The code used to derive the score and generate the figures is available from the corresponding author on reasonable request.

## Abbreviations

ADL: activities of daily living
ALT: alanine aminotransferase
AST: aspartate aminotransferase
BMI: body-mass index
BP: blood pressure
C: concordance statistic
CARRS: Centre for cArdiometabolic Risk Reduction in South Asia
CHARLS: China Health and Retirement Longitudinal Study
CI: confidence interval
CKD-EPI: Chronic Kidney Disease Epidemiology Collaboration
DCA: decision-curve analysis
DSM-5: Diagnostic and Statistical Manual of Mental Disorders,5th edition
eGFR: estimated glomerular filtration rate
HbA1c: glycated haemoglobin
HMSE: Hindi Mental State Examination
HR: hazard ratio
hs-CRP: high-sensitivity C-reactive protein
IADL: instrumental activities of daily living
IQR: interquartile range
LASI: Longitudinal Aging Study in India
LASI-DAD: Longitudinal Aging Study in India – Diagnostic Assessment of Dementia
MCI: mild cognitive impairment
NLR: neutrophil-to-lymphocyte ratio
NT-proBNP: N-terminal pro-B-type natriuretic peptide
SAGE: Study on global AGEing and adult health
SD: standard deviation
T3: triiodothyronine
TRIPOD: Transparent Reporting of a multivariable prediction model for Individual Prognosis Or Diagnosis
ULSAM: Uppsala Longitudinal Study of Adult Men
WHO: World Health Organization
